# Chromosome-Level Genome Assembly and HazelOmics Database Construction Provides Insights Into Unsaturated Fatty Acid Synthesis and Cold Resistance in Hazelnut (*Corylus heterophylla*)

**DOI:** 10.3389/fpls.2021.766548

**Published:** 2021-12-09

**Authors:** Jianfeng Liu, Heng Wei, Xingzheng Zhang, Hongli He, Yunqing Cheng, Daoming Wang

**Affiliations:** ^1^Jilin Provincial Key Laboratory of Plant Resource Science and Green Production, Jilin Normal University, Siping, China; ^2^Liaoning Economic Forest Research Institute, Dalian, China

**Keywords:** *Corylus heterophylla*, genome assembly, PacBio sequencing, Hi-C, unsaturated fatty acid, cold resistance

## Abstract

*Corylus heterophylla* (2n = 22) is the most widely distributed, unique, and economically important nut species in China. Chromosome-level genomes of *C. avellana*, *C. heterophylla*, and *C. mandshurica* have been published in 2021, but a satisfactory hazelnut genome database is absent. Northeast China is the main distribution and cultivation area of *C. heterophylla*, and the mechanism underlying the adaptation of *C. heterophylla* to extremely low temperature in this area remains unclear. Using single-molecule real-time sequencing and the chromosomal conformational capture (Hi-C) assisted genome assembly strategy, we obtained a high-quality chromosome-scale genome sequence of *C. heterophylla*, with a total length of 343 Mb and scaffold N50 of 32.88 Mb. A total of 94.72% of the test genes from the assembled genome could be aligned to the Embryophyta_odb9 database. In total, 22,319 protein-coding genes were predicted, and 21,056 (94.34%) were annotated in the assembled genome. A HazelOmics online database (HOD) containing the assembled genome, gene-coding sequences, protein sequences, and various types of annotation information was constructed. This database has a user-friendly and straightforward interface. In total, 439 contracted genes and 3,810 expanded genes were identified through genome evolution analysis, and 17 expanded genes were significantly enriched in the unsaturated fatty acid biosynthesis pathway (ko01040). Transcriptome analysis results showed that *FAD* (Cor0058010.1), *SAD* (Cor0141290.1), and *KAT* (Cor0122500.1) with high expression abundance were upregulated at the ovule maturity stage. We deduced that the expansion of these genes may promote high unsaturated fatty acid content in the kernels and improve the adaptability of *C. heterophylla* to the cold climate of Northeast China. The reference genome and database will be beneficial for future molecular breeding and gene function studies in this nut species, as well as for evolutionary research on species of the order Fagales.

## Introduction

Hazelnut (*Corylus* spp.) belongs to the subfamily Coryloidae and is the most widely distributed and economically important genus in the Betulaceae family (Helmstetter et al., 2019). The fruit of the hazelnut, the hazelnut, is rich in nutrients and fatty acids and is widely used in the food industry. Its uses include making oil, paste, and roasted kernels (Madhaven, 2000; Amaral et al., 2006). In addition, crushed kernels are used in the production of cakes, ice cream, and chocolate to improve the flavor, and these products are widely popular with consumers. There are approximately 16 species of hazelnut in the world, ten of which are native to China, comprising eight wild species and two cultivated species (Dong et al., 2010). Among these, *Corylus heterophylla* is the most widely distributed and economically important nut species in China. Currently, the area of *C. heterophylla* forests in China covers more than 1.0 million hectares, and this species is the main source of hazelnut in the Chinese market even though the yield of hybrid hazelnut (*C. heterophylla* × *C. avellana*) has increased rapidly in recent years (Cheng et al., 2018b,^2019^; Liu et al., 2020). Northeast China is the main distribution and cultivation area of hazelnut. Although most hazelnut products in the international market are derived from the European hazelnut (*C. avellana*), the extreme low winter temperatures in Northeast China are not suitable for the cultivation of this species. Although the area of horticultural cultivation of *C. heterophylla* is increasing rapidly, its commercial varieties remain scarce. As *C. heterophylla* is a unique species of hazelnut in China, reasonable utilization of its germplasm is crucial for the development of the hazelnut industry. Therefore, genome analysis of *C. heterophylla* would be important to provide new insights into the key adaptations that contribute to the breeding and culture of *C. heterophylla*.

In 2013, to facilitate breeding and genetic studies on hazelnut, the genome sequence of *C. avellana* ‘Jefferson’ was assembled and released online on the European Hazelnut Genomic Resource Portal (EHG^1^) (Sathuvalli et al., 2011; Rowley et al., 2018), but the structural and functional annotations of its genes are not available. EHG plays an important role in genetic studies on hazelnut by providing the scientific community access to the genome sequence of the variety ‘Jefferson’. However, *C. avellana* is not an important cultivated species, although some cultivars have been introduced and cultured in China. EHG still needs further improvement because it lacks the structural and functional annotations of genes, which are vital for gene bioinformatics of the hazelnut genome. During the past few years, some genome re-sequencing data and transcriptome data of hazelnut, including *C. heterophylla* × *C. avellana* and *C. heterophylla*, have accumulated at an increasing rate (Rowley et al., 2012; Chen et al., 2014; Cheng et al., 2018b,^2019^; Liu et al., 2020). In 2021, high-quality chromosome-level reference genomes for *C. heterophylla* (Zhao et al., 2021) and *C. mandshurica* (Li et al., 2021) based on combining Illumina short reads, Nanopore long reads, and chromosomal conformational capture (Hi-C) sequencing reads were published, thus providing a valuable resource for hazelnut breeding. However, a satisfactory hazelnut genome database is absent, hindering hazelnut breeding research. Considering the economic importance of *C. heterophylla* in the main production areas of Northeast China and the shortage of its cultivars, it is crucial to construct a hazelnut genome database to archive and access genome sequences of this species. Here, we present a complete reference genome sequence and the constructed HazelOmics online database of *C. heterophylla* with the aim of providing mass data of high-class reference genomes and evolution of this important nut species.

## Materials and Methods

### Plant Sample Collection and Genome Sequencing

*Corylus heterophylla* was cultivated in Yitong County of Siping City (43.34°N, 125.30°E), Jilin Province, China. Fresh young leaves of *C. heterophylla* were collected and subjected to genomic DNA extraction, PacBio genome sequencing library construction, and single molecule real-time (SMRT) sequencing. The template library was constructed using the SMRTbell Template Prep Kit 1.0 (product code 100-259-100); the experimental steps are as follows. Leaf cells were lysed, and genomic DNA was sheared by the Covaris g-Tube, followed by exonuclease VII digestion to remove the single chain at the 3′-end. Next, the SMRTbell Damage Repair Kit was used to repair single-strand breaks, base loss, and oxidation on the DNA strand. DNA was repaired to a flat end by terminal repair, and DNA fragments were connected with the SMRT dumbbell connector. Thereafter, exonuclease digestion was performed to remove the fragments without SMRT dumbbell connector at both ends. Finally, the AMPure ^®^ PB Beads were used for secondary screening and purification to obtain an SMRTbell library with a fragment size of 20 kb. The library was sequenced using the long-read PacBio Sequel II platform (Pacific Biosciences Inc., Menlo Park, CA, United States), and data from one SMRT cell were generated. Second-generation survey sequencing was performed to provide sequence information for error correction of the assembled genome based on SMRT sequencing. DNA was isolated using the DNeasy Plant Mini Kit (Qiagen, Valencia, CA, United States) according to the manufacturer’s recommendations. DNA purity was evaluated by a Nanodrop spectrophotometer (Thermo Fisher Scientific, Wilmington, DE, United States) and Qubit 2.0 Fluorometer (Thermo Fisher Scientific Inc., Waltham, MA, United States), and DNA integrity was evaluated by electrophoresis. The qualified DNA sample was fragmented randomly using a g-tube. Fragments ranging from 300 to 350 bp in length were recycled by electrophoresis, and the sequencing library was prepared by terminal repair, addition of an A tail, addition of a sequencing adaptor, purification, and PCR amplification, followed by sequencing on the Illumina HiSeq X Ten platform (San Diego, CA, United States) with 150PEmode. All sequencing procedures were performed by Wuhan GOOAL Gene Technology Co., Ltd.

### Hi-C Sequencing

The Hi-C library for Illumina sequencing was prepped by the NEBNext ^®^ Ultra™ II DNA library Prep Kit for Illumina (NEB) according to the manufacturers’ instructions. First, genomic DNA was treated with paraformaldehyde to fix the DNA conformation, and the cross-linked DNA was treated with restriction enzymes to produce sticky ends. At the same time, biotin was introduced to label the oligonucleotide ends. Thereafter, DNA ligase was used to connect the DNA fragments. DNA cross-linking was reversed by protease digestion, after which the DNA was purified and randomly broken into 300–500 bp fragments using Covaris E220 Evolution Sonicator (Woburn, MA, United States). Finally, the labeled DNA was captured by avidin magnetic beads and used to construct a Hi-C sequencing library with the NEBNext ^®^ Ultra™ II DNA library Prep Kit, followed by sequencing on the Illumina HiSeq X Ten platform with 150PEmode.

### Genome Assembly

Canu software (Koren et al., 2017) was used to assemble the acquired raw reads. The assembly contained three steps: error correction, trimming, and assembly, and each step was carried out using the following processing protocol. Reads were loaded to the gkpStore read database, and k-mers were counted to evaluate overlaps between sequences. Overlaps were loaded to the overlap database OvlStore to complete error correction, trimming, or assembly. Details and parameter information of all used software are listed in Supplementary Table 1. Single molecule real-time sequencing has a high error rate, which makes the original very noisy. In the process of correction, highly reliable bases are obtained by comparing the reads. Consistent sequences were obtained by calculating the overlapped reads, which were used to replace the original reads with high error rates. In the process of read trimming, overlap was used to determine which read regions were of high quality and which low-quality regions needed to be trimmed; only sequence blocks with the highest quality were retained. Next, the original offline data of SMRT sequencing were mapped to the assembled genome for error correction analysis using pbmm2 software, and the corrected assembled genome was generated after polishing using the arrow method. Thereafter, the reads obtained from Illumina genome survey were mapped to the third generation assembled genome for further polishing using BWA (Li and Durbin, 2009) and Pilon (Walker et al., 2014) software for sequence alignment and error correction, respectively. According to the depth distribution of reads and sequence similarity, redundant heterozygous contigs were identified and removed using Purge Haplotigs software (Roach et al., 2018).

### Chromosome-Scale Assembly With Hi-C Data

The main types of reads produced by Hi-C sequencing data comprise valid di-tags, contiguous sequences, circularized fragments, dangling ends, internal fragments, PCR duplicates, and wrong sized reads. We retained only the valid di-tags, and other types of reads were filtered out (Dudchenko et al., 2017). Chromosome-level genome assembly was carried out by dividing, anchoring, sequencing, orienting, and merging the contigs or scaffolds using HiC-Pro (Servant et al., 2015; Dudchenko et al., 2017). A genome-wide interaction map was constructed using JuiceBox software (Durand et al., 2016). We encountered contig sequencing and orientation errors in the process of 3D-DNA assembly. According to the principle that the closer the linear distance, the stronger the interaction, we carried out visualized error correction manually using JuiceBox (Durand et al., 2016). The integrity assessment of conserved genes from the assembled genome was assessed using Benchmarking Universal Single-Copy Orthologs (BUSCO) tests (Simao et al., 2015), and its results reflected the completeness and quality of the test genome. In total, 1,440 single-copy orthologous genes were chosen to be aligned to the Embryophyta_odb9 database (Simao et al., 2015).

### Genome Annotation

Genome annotation analysis mainly includes the recognition of repetitive sequences, prediction of non-coding RNA, prediction of gene structure, and functional annotation. As an important part of the plant genome, repeat sequences mainly include tandem repeats and interspersed repeats (DNA transposons and retrotransposons). Tandem Repeats Finder software (Benson, 1999) was used to predict tandem repeats in the investigated genome. RepeatMasker and RepeatProteinMask based on Repbase TE library were used to acquire the annotation of DNA transposons and retrotransposons (Tarailo-Graovac and Chen, 2009). Afterward, *de novo* prediction software RepeatModeler (Bao et al., 2015) and LTR_FINDER (Barker et al., 2010) were used to identify and annotate interspersed repeats in the hazelnut genome.

Gene annotation of hazelnut includes structural and functional annotation. Several methods were used to predict the structure of the coding genes, such as homology prediction, *de novo* prediction (software: Augustus, GENSCAN, and GlimmerHMM), and cDNA/EST prediction (Burge and Karlin, 1997; Majoros et al., 2004; Mario et al., 2006). Furthermore, RNA-seq data were mapped to genome by HISAT2 (Kim et al., 2015) and transcripts were generated by StringTie (Pertea et al., 2015). After that, Transdecoder (Haas et al., 2013) was used to predict ORF in these transcripts. The gene set predicted by various methods was integrated into a non-redundant, more complete, and reliable gene set using MAKER software (Cantarel et al., 2008). Finally, functional annotation of the proteins in the investigated gene set was carried out by aligning their protein sequences to various protein databases, including SwissProt (Bairoch and Apweiler, 2000), TrEMBL (Bairoch and Apweiler, 2000), Kyoto Encyclopedia of Genes and Genomes (KEGG) (Kanehisa et al., 2003), InterPro (Zdobnov and Rolf, 2001), and Gene Ontology (GO) (Balakrishnan et al., 2013). For non-coding RNA annotation, tRNAscan-SE program (Lowe and Chan, 2016) was used to identify tRNA, BLASTN alignment was used to identify rRNA, and INFERNAL software (Nawrocki et al., 2009) of the Rfam database (Griffiths-Jones et al., 2005) was used to predict miRNA and snRNA sequences in the genome.

### Gene Family and Phylogenetic Analyses

The longest transcript of each gene in each species was obtained by an in-house script as the representative sequence of the gene, and their coding sequence (CDS) and protein sequence information was obtained. The version and other details of all downloaded sequences are listed in Supplementary Table 2. The homologous low copy genes (copy number ≤ 4) of these species were identified by orthofinder software (Emms and Kelly, 2015). OrthoMCL software and Markov chain clustering (MCL) (Li et al., 2003) were used to evaluate gene family membership based on obtained gene similarity calculation results. The protein sequences of these low copy homologous genes were aligned by muscle software, and the phylogenetic tree was constructed by RAxML software based on the results of multiple sequence alignment using the GTRGAMMA method (Stamatakis, 2014). Next, according to the results of the phylogenetic tree, r8s (Sanderson, 2003) and MCMCTREE of the PAML package (Yang, 2007) were used to estimate divergence time. The divergence times of *Oryza sativa*–*Arabidopsis thaliana* [115–308 million years ago (Mya)], *Betula pendula*–*C. avellana* (22–74 Mya), and *Populus trichocarpa*–*P. euphratica* (10.9 Mya) acquired from TimeTree^2^ were used as the calibration times. Gene families that underwent expansion or contraction events were identified by CAFE software (Han et al., 2013). The identified genes were subjected to further analysis of GO term enrichment and KEGG enrichment, and the p-value of significant enrichment was set as 0.05 in GO term and KEGG enrichment analysis (Kanehisa et al., 2003; Balakrishnan et al., 2013).

### Construction of HazelOmics Online Database

The HazelOmics online database (HOD) was constructed based on the assembled *C. heterophylla* reference genome. The establishment and maintenance of HOD was entrusted to GOOAL GENE Technology Ltd. (Wuhan, China) and the Information Network Center of the Jilin Normal University. For online website building, the website interface was developed based on the Vue.JS framework. Three universally used open source application framework or database management systems, Spring Boot, JDK8, and MySQL, were employed for database server development to facilitate user access and operation. In addition, the genome data stored in HOD can be visualized by JBrowse and its plugins (Buels et al., 2016). A sequence query option was also added to the website using the BLAST tool. The Primer3 tool (Rozen and Skaletsky, 2000) was provided for primer design. All available sequences, along with corresponding function annotation information (genomes, protein-coding sequences, and protein sequences), can be downloaded from HOD.

### RNA-seq Analysis and KEGG Pathway Enrichment of Differentially Expressed Genes

RNA-seq analysis of 12 ovule samples of hazelnut at four developmental stages was performed following the protocol described previously (Liu et al., 2020). HISAT2 (Kim et al., 2015) was used to align the transcripts to our reference genome, and a sam file was generated. Samtools (Li et al., 2009) software was used to convert the obtained Sam file into BAM format. After sorting, qualimap software (Okonechnikov et al., 2016) was used to count the sequence alignment results. The transcripts were assembled according to BAM files by StringTie software (Pertea et al., 2015), and the reconstructed results of all samples were merged to obtain the structure annotation file of the optimized transcripts. Gene expression was quantified according to the BAM file using StringTie (Pertea et al., 2015) and expressed in FPKM values. | log_2_FC | ≥ 1 and false discovery rate < 0.05 were set as the threshold values for the identification of differentially expressed genes. KEGG enrichment analysis was performed using KOBAS software (Xie et al., 2011).

## Results

### Single Molecule Real-Time Sequencing and *de novo* Genome Assembly

A total of 7,319,564 reads were sequenced, among which 6,803,436 reads were longer than 2.0 kb, accounting for 92.94% of all sequenced reads. The sequencing generated 144.01 Gb of PacBio sequencing data from the SMRT sequencing platform, achieving ∼415 × coverage of the *C. heterophylla* genome (Table 1). On average, the reads were 19,675 bp in length, with N50 of 30,570 bp. These results suggested that SMRT sequencing is reliable and can produce long reads (Vaser et al., 2017; Vasanthan and Yasubumi, 2019). The read sequences were assembled by Canu (Koren et al., 2017). Next, the original offline SMRT sequencing data and second-generation survey sequencing were mapped to the assembled genome for error correction, followed by redundant sequence removal using Purge Haplotigs software (Roach et al., 2018). The genome assembly analysis produced 386 contigs with N50 of 2,025,119 bp and GC content of 36.01%, covering 346,578,452 bp. Among the contigs, 384 were longer than 2 kb (Table 1). To confirm that the obtained assembly belongs to the target species, the genomic sequence was divided into 1,000 bp fragments, and the divided sequence was aligned to the NCBI nucleotide database (NT Library) using the Blast tool. The results showed that 10.20% of the fragments belonged to the genus *Corylus*, and the accuracy of the sequencing and assembly data was preliminarily confirmed (Supplementary Table 3). In addition, integrity assessment of conserved genes was performed using the method of BUSCO (Simao et al., 2015). Of the chosen 1,440 single-copy orthologous genes, 1,364 (94.72%) were aligned to the Embryophyta_odb9 database (Simao et al., 2015), of which 1,338 (92.92%) were considered to be complete (Supplementary Table 4). The SMRT sequencing data were mapped to the assembled genome using pbmm2 software program; the results showed that 92.46% of SMRT data could be mapped to the assembled genome with coverage of 99.72%, suggesting high quality of genome assembly (Table 2). In summary, the contigs of the assembled genome can be extended to the scaffold by downstream analysis up to the chromosome level.

**TABLE 1:** Statistical results of *C. heterophylla* genome sequencing and assembly.

	Total bases (Gb)	Total length (bp)	Total number	Total number (≥ 2 kb)	Max length (bp)	Mean length (bp)	N50 (bp)	N90 (bp)	GC content (%)
Library	144.01	–	7,319,564	6,803,436	238,716	19,675	30,570	11,171	37.9
Assembly	–	346,578,452	386	384	7,707,050	–	2,025,119	424,534	36.01

**TABLE 2:** Sequences consistency assessment by comparing with the *C. heterophylla* reference genome.

Sequencing platform	Mapping rate (%)	Paired mapping rate (%)	Coverage (%)	Coverage at least 4 × (%)	Coverage at least 10 × (%)	Coverage at least 20 × (%)
PacBio	92.46	–	99.72	99.54	99.32	99.13
Hi-C	91.04	84.89	98.97	98.67	98.27	97.66

### Hi-C Sequencing and Assisted Genome Assembly

**FIGURE 1:**
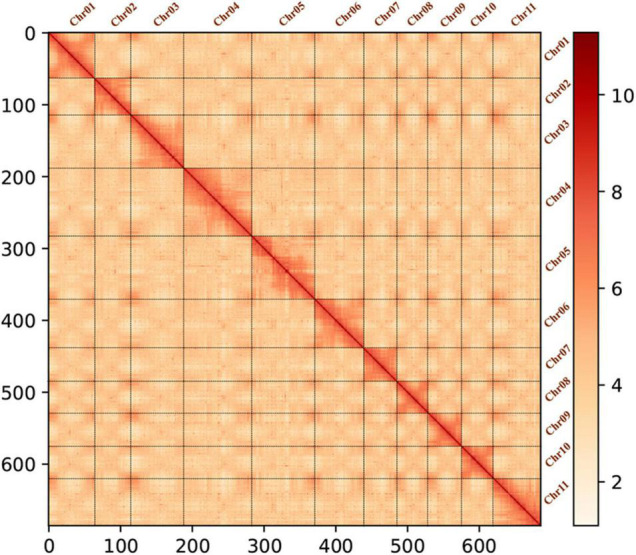
Hi-C heatmap of the *C. heterophylla* genome representing genome-wide all-by-all interactions. The map shows a high-resolution of individual chromosomes that are scaffolded and assembled independently. The color bar depicts the frequency of Hi-C interaction links from white (low) to red (high). The coordinates on the x- and y-axes can be used to determine the relative position (number of bins) on the genome.

In total, 392,233,610 raw reads were sequenced, covering a length of 58,835,041,500 bp. After data filtration, 383,274,912 clean reads covering 56,648,423,236 clean bases were obtained, with average read length of 150 bp and Q20 of 96.24% (Supplementary Table 5). The Hi-C sequencing data were mapped to the assembled genome using BWA (Li and Durbin, 2009) software program, and the results showed that 91.04% of Hi-C data could be mapped to the assembled genome, with coverage of 98.97% (Table 2). Alignment results of Hi-C sequencing data showed that 84,085,237 reads belonged to paired-end alignments (Supplementary Table 6). After redundancy removal, valid pairs accounted for 73.72% of the total Hi-C sequencing data (Supplementary Table 7). Mutations were identified by Samtools, Picard, and GATK software programs (Li et al., 2009; do Valle et al., 2016), and the homozygous and heterozygous rate of SNPs and indels of the reference genome were calculated. The homozygous rate of SNPs and indels were as low as 0.011% and 0.037%, respectively, which indicated that the accuracy of the genome assembly was very high; the heterozygous rate of SNPs and indels were as low as 1.118% and 0.216%, respectively, indicating that genome heterozygosity was low (Supplementary Table 8). In the process of assembly and error correction, the original 386 contigs were split and sorted according to the Hi-C interaction map, and 11 chromosomes and 64 scaffolds were constructed, with a total length of 0.35 Gb, contig N50 of 2.02 Mb, and scaffold N50 of 32.88 Mb. The rate of chromosome anchoring was 98.95% (Table 3). Genome integrity was evaluated using long terminal repeats (LTRs) (Ou et al., 2018). The LTR assembly index (LAI), a standard for assessing assembly continuity, of the genome was 14.20, which was within the range of “reference” quality based on the LAI classification (Supplementary Table 9) (Ou et al., 2018; Xie et al., 2020). Subsequently, chromosome and genome-wide interaction maps (Figure 1) were constructed, and the results showed that the Hi-C assisted assembly was of high quality. Finally, based on the reference genome generated by SMRT sequencing and assembly (Jonas et al., 2017), Hi-C sequencing (Servant et al., 2015) and assisted genome assembly were further performed to correct the errors in the genome, and the final genome sequence and direction of *C. heterophylla* was determined. The length of the final assembled genome was 342,961,297 bp, with contig N50 of 2,025,119 bp, scaffold N50 of 32,881,252 bp, and chromosome anchoring rate of 98.95% (Table 3).

**TABLE 3:** Statistic for Hi-C auxiliary genome assembly.

	Sequence length (bp)	Sequence number	Contig N50 (bp)	Scaffold N50 (bp)	Chromosome anchoring rate (%)			
					Contig number	Contig length (bp)	Contig number (> 100 kb)	Contig length (> 100 kb)
Draft genome	346,578,452	386	2,025,119	2,025,119	–	–	–	
Genome assembly (+ Hi-C)	346,614,552	75	2,017,784	32,881,252	83.49	98.95	96.83	99.38
Chromosome assembly (+ Hi-C)	342,961,297	11	2,025,119	32,881,252	–	–	–	
Unanchored sequences (+ Hi-C)	3,653,255	64	114,029	190,000	–	–	–	

### Genome Annotation

In total, we identified 200.99 Mb of non-redundant repetitive elements based on *de novo* and homolog methods, accounting for 57.99% of the assembled genome length (Table 4). Among these, long terminal retrotransposons (LTR-RTs), DNA transposons, and long interspersed elements (LINEs) had lengths of 110.26, 68.48, and 30.42 Mb, accounting for 31.81%, 19.76%, and 8.78% of the total genome length, respectively. This suggested that LTR-RTs dominated the repetitive sequences in the investigated genome. Based on *de novo*, homolog, and RNA-seq/EST methods, we predicted a total of 22,319 protein-coding genes in the investigated genome, with the average length of 6,646 bp. Average CDS length was 1,201 bp. On average, each gene had 5.61 exons that were 330 bp long. Average intron length was 1,040 bp (Supplementary Table 10). Among the predicted protein-coding genes, 14,763 (66.15%) genes were supported by evidence of *de novo* prediction, homologous prediction, and RNA-seq data (Supplementary Table 11). In total, 21,056 (94.34%) predicted genes had matching functional annotations in at least one protein database (Supplementary Table 12). In total, 92.7% of the conserved single-copy orthologs used by BUSCO could be mapped to the Embryophyta_odb9 database (Simao et al., 2015), of which 93.1% were complete (Supplementary Table 13). In addition, we further annotated the non-coding RNAs covering the length of 346,578,452 bp and accounting for 0.13% of the total genome. The predicted non-coding RNAs included 453 tRNA genes, 1,020 rRNA genes, 183 miRNA genes, and 595 snRNA (Table 5).

**TABLE 4:** Category of repeat sequences in *C. heterophylla* genome.

Categories	RepBase TEs		TE Proteins		*De novo*		Combined TEs	
	Length (bp)	Percentage in genome (%)	Length (bp)	Percentage in genome (%)	Length (bp)	Percentage in genome (%)	Length (bp)	Percentage in genome (%)
DNA	9,799,717	2.83	3,919,867	1.13	62,545,620	18.05	68,475,106	19.76
LINE	7,164,463	2.07	8,921,980	2.57	27,339,957	7.89	30,417,364	8.78
SINE	24,749	0.01	0	0	27,221	0.01	51,884	0.01
LTR-RT	23,038,666	6.65	16,976,559	4.9	106,305,286	30.67	110,258,874	31.81
Satellite	229,854	0.07	0	0	550,649	0.16	696,377	0.2
Simple_repeat	0	0	0	0	2,587,539	0.75	2,587,539	0.75
Other	1,456	0	0	0	0	0	1,456	0
Unknown	150,029	0.04	10,428	0	23,266,035	6.71	23,410,828	6.75
Total	39,297,124	11.34	29,823,067	8.6	191,137,480	55.15	200,988,864	57.99

**TABLE 5:** Annotation statistics for *C. heterophylla* genome.

Type		Copy	Average length (bp)	Total length (bp)	Percentage (%)
miRNA		183	125	22,798	0.006578
tRNA		453	75	34,003	0.009811
rRNA	rRNA	1,020	127	129,161	0.037267
	18S	7	1,228	8,594	0.00248
	28S	4	187	748	0.000216
	5.8S	8	155	1,242	0.000358
	5S	1,001	118	118,577	0.034214
snRNA	snRNA	595	114	67,860	0.01958
	CD-box	408	105	42,759	0.012337
	HACA-box	58	125	7,233	0.002087
	splicing	129	139	17,868	0.005156
	scaRNA	0	0	0	0

### Phylogenetic Relationship Analysis

**FIGURE 2:**
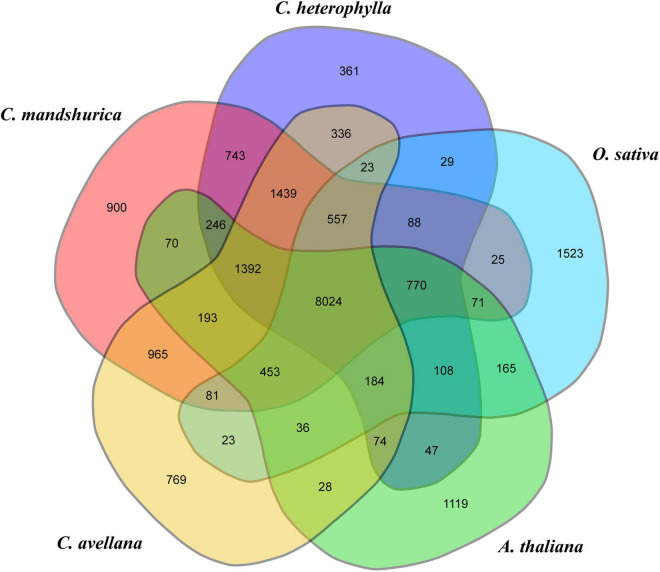
Comparative genomic analysis of *C. heterophylla* and other species. Venn diagram representing the cluster distribution of shared gene family among *C. heterophylla* and four other species, including *A. thaliana*, *O. sativa*, *C. avellana*, and *C. mandshurica*.

**FIGURE 3:**
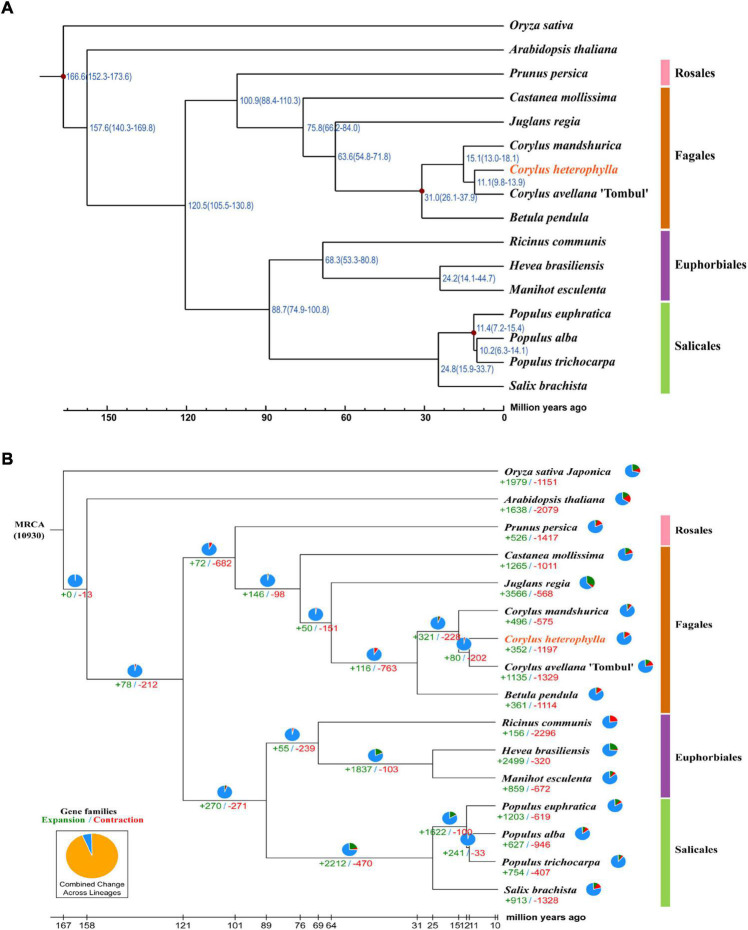
Phylogenetic and evolutionary analysis of the *C. heterophylla* genome. Among all used species, members belong to Rosales, Fagales, Euphorbiales, and Salicales are indicated in pink, orange, purple, and green, respectively. **(A)** The phylogenetic tree is constructed based on a concatenated alignment of 695 single-copy ortholog gene sets. The estimated divergence times (million years ago, MYA) are indicated at each node. The reference points used for calibration are marked with red dots. **(B)** Expansions and contractions of gene families are indicated in green and red, respectively. The pie charts show the proportions of conserved (blue), expanded (green) and contracted (red) gene families among all gene families.

Because of the limited number of species in the order Fagales, the annotated genes in the investigated genome were clustered into gene families with 15 closely related species with available genome information, comprising two model plant species (*O. sativa* and *A. thaliana*), five species from the order Fagales (*C. avellana*, *C. mandshurica*, *B. pendula*, *Castanea mollissima*, and *Juglans regia*), three species from the order Euphorbiales (*Ricinus communis*, *Manihot esculenta*, and *Hevea brasiliensis*), four species from the order Salicales (*Salix brachista*, *Populus alba*, *P. trichocarpa*, and *P. euphratica*), and one species from the order Rosales (*Prunus persica*) (Supplementary Table 2). OrthoMCL gene family clustering analysis revealed that 19,683 *C. heterophylla* genes (88.19%) clustered into 14,421 gene families, and 113 of these were specific for *C. heterophylla* (Supplementary Table 14). In addition, the results of this analysis showed that *C. heterophylla*, *A. thaliana*, *O. sativa*, *C. avellana*, and *C. mandshurica* shared a core set of 8,024 gene families (Figure 2). Furthermore, after OrthoMCL clustering, 695 single-copy gene families were selected from the 16 analyzed species for subsequent analysis. Genetic evolutionary analysis revealed that *C. heterophylla* was a sister group to *C. avellana*, and the estimated divergence time between them was 11.1 (9.8–13.9) million years ago (Figure 3A). Our phylogenetic tree suggested that the species from the same order have a close genetic relationship, and the relationships between the 16 investigated species were consistent with their taxonomic positions and the results of previous phylogenetic analyses (Flora and Sciences, 1979; Cheng et al., 2018a).

### The Expansion and Contraction of Gene Families

**FIGURE 4:**
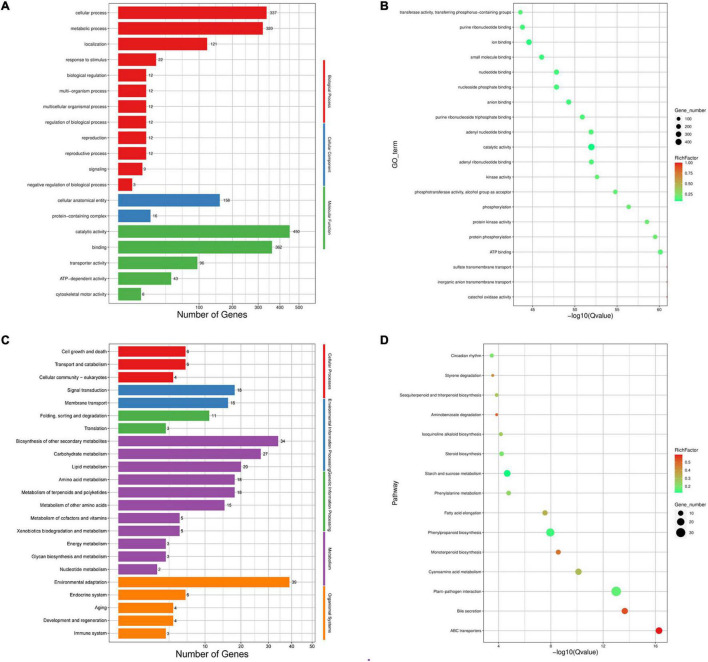
Gene ontology (GO) and KEGG enrichment of genes belonged to expansion and contraction gene families in *C. heterophylla* genome. **(A,B)** Represent the GO enrichment of genes; **(C,D)** represent the KEGG enrichment of genes.

The expansion and contraction of gene families play critical roles in driving the phenotypic diversification of plants. We identified 352 expanded and 1,197 contracted gene families in *C. heterophylla* relative to *C. avellana* (Figure 3B). Gene Ontology classification analysis of expanding and contracting genes suggested that the most abundant genes were related to cellular, metabolic, and localization processes, and they were mainly located in membrane and intracellular organelles of cellular anatomical entity and executed molecular functions of catalytic activity and binding (Figure 4A). Gene Ontology enrichment analysis indicated that multiple GO terms were significantly enriched, including inorganic anion and sulfate transmembrane transport, ATP binding, protein phosphorylation, and catalytic activity (Figure 4B). KEGG enrichment analysis revealed multiple significantly enriched KEGG pathways, including fatty acid elongation and plant–pathogen interaction (Figures 4C,D). These results may indicate significant differences in fatty acid biosynthesis and environmental adaptation between *C. heterophylla* and *C. avellana*.

### Construction of the HazelOmics Database

In order to facilitate future genetic studies and molecular breeding of hazelnut, the HazelOmics Database (HOD^3^) was constructed based on genome sequencing and assembling of the Chinese cultivar *C. heterophylla* ‘Jizhen 6.’ The database includes the assembled genome sequences, predicted genes, CDS, and protein sequences, along with their functional annotations. The server was explored using several tools and software programs with a user-friendly and straightforward interface, including Spring Boot, JDK8, and MySQL. Locations where hazels were planted and collected were marked on the map on the website homepage. HOD offers four major homepage sections or entries for the users to choose from, named JBrowse (Buels et al., 2016), BLAST, Primer design, and Download. Detailed species and genome data are presented in the Genome subsection. The widely used genome browser JBrowse (Buels et al., 2016) was employed for displaying genome sequences, gene positions, and structures. To provide a gene retrieval function in HOD, we used the embedded BLAST sequence server tool. All known sequences of hazelnut, including genome, CDS, and protein sequences stored in HOD, are available for alignment using the BLAST program. Users are permitted to retrieve and download the genome, CDS, and protein sequences along with their corresponding annotation files in GFF3 format. Conveniently, an entry with the embedded Primer 3 tool is also offered on the website homepage for primer design.

**FIGURE 5:**
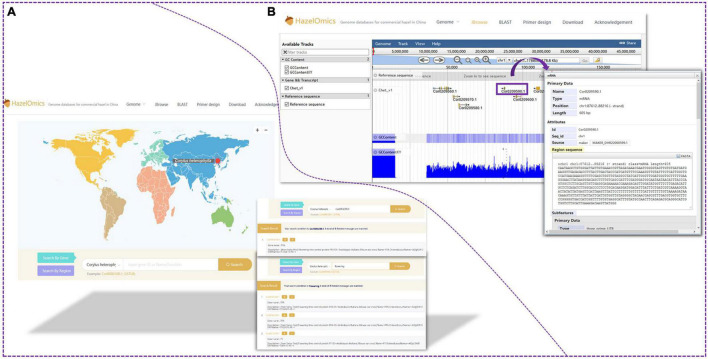
Gene search and JBrowse functions. **(A)** The database provided an interface for querying with gene ID, name or function in genes search option. **(B)** Users are also able to visualize the location of genes in the genome with the help of JBrowse.

**FIGURE 6:**
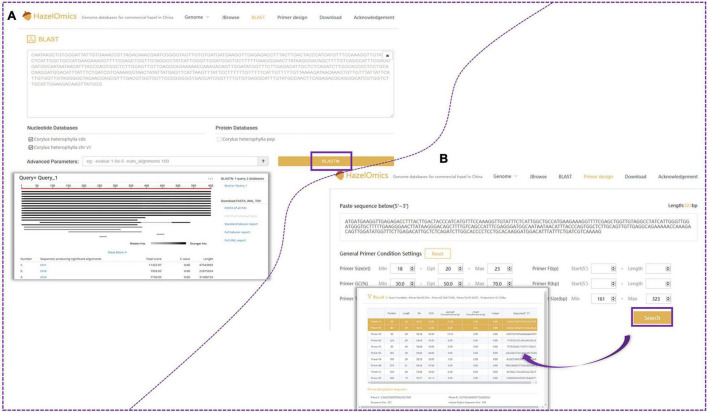
Blast and primer design function. **(A)** Users can search for *C. heterophylla* homologous genes by using Blast function. **(B)** Users also can design primers using the embedded “Primer 3” tool. Several parameters of anticipant primers for target sequence, containing primer size (nt), GC content (%) and primer Tm (°C), can be users defined.

For gene and genome region search, two search options and entries are provided on the homepage: “Search by gene” and “Search by region.” Furthermore, the webpage provides an interface for querying gene ID, name, or function in the “gene search option” (Figures 5A,B). For sequence alignment and homology queries, the Blast program was embedded in HOD, and the entry for sequence blasting is also available on the website homepage (Figure 6A). The parameters for filtering low-homology sequences of the returned blast hits can be manually set based on user demands. Users can provide all available sequences (such as genome, transcript, CDS, and protein sequences) in the textbox or upload the sequence file for homology query by comparison with sequences stored in HOD, and query results will be sorted according to the blast scores or E-value on the results page and can be downloaded in FASTA, XML, or TSV format. All hits are also linked to a graphic output with detailed information, including the sequence alignment sketch map, blast E-value, and identities between query and hit sequences. In HOD, a “Primer design” option is also embedded in the main menu on the homepage, which can conveniently be used for subsequent molecular research (Figure 6B). The usage and fundamental principles of this option are similar to those of the mainstream software or tools for primer design, such as Primer Premier 5. Several parameters of predicted primers for the target sequence, containing primer size (nt), GC content (%), and primer Tm (°C), can be defined by the user. All theoretically usable primer pairs will be listed in the results page, and they will be comprehensively ordered by primer quality considering several parameters, including primer GC content, Tm, any or 3′ self-complementarity, and hairpin.

### Expanding Genes Related to Unsaturated Fatty Acids Biosynthesis in the Hazelnut Genome

Hazelnut kernels are rich in fatty acids. Analysis of gene expansion and gene contraction in *C. heterophylla* and its related species revealed that the expansion and contraction of *C. heterophylla* genes were significantly enriched in the KEGG pathway of fatty acid biosynthesis, which indicated that changes in fatty acid gene families led to the formation of special adaptive mechanisms in *C. heterophylla*. To further understand the peculiarity of fatty acid synthesis in *C. heterophylla*, we analyzed the genome evolution of *C. heterophylla* together with 14 other important oil plants, including *Arachis duranensis*, *A. thaliana*, *Brassica napus*, *C. avellana*, *Camellia sinensis*, *Glycine max*, *Juglans regia*, *Ostryopsis davidiana, Olea europaea*, *Ostryopsis intermedia*, *Ostryopsis nobilis*, *O. sativa*, *Sesamum indicum*, *and Zea mays* (Supplementary Table 2). In total, 1,277 unique family genes were found in *C. heterophylla*, belonging to 1,272 unique families (Supplementary Table 15), which may be related to the specificity of *C. heterophylla*. Random occurrence and death patterns were used to simulate the expansion and contraction events of gene families in each lineage of the evolutionary tree, and 439 contracted genes and 3,810 expanded genes were identified. These genes were further subjected to KEGG enrichment analysis (Supplementary Tables 16, 17). We focused on the differences in biological characteristics of fatty acid synthesis between *C. heterophylla* and other oil plants. A total of 30 genes related to the synthesis of unsaturated fatty acids were identified in the genome of *C. heterophylla*, and 17 expanded genes were significantly enriched in the biosynthesis of the unsaturated fatty acids pathway (ko01040). Therefore, compared with the 14 other oil plants mentioned above, *C. heterophylla* was unique in unsaturated fatty acid synthesis, which is consistent with the extremely high unsaturated fatty acid level in the kernels of *C. heterophylla* (Song et al., 2008).

**FIGURE 7:**
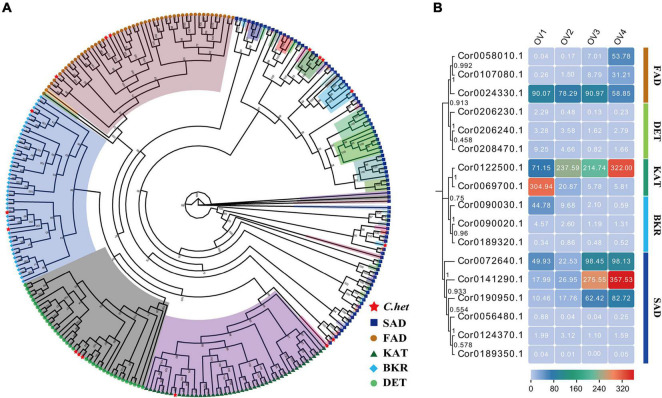
Identification and expression analyses of expanded genes in biosynthesis of unsaturated fatty acids pathway (ko01040). **(A)** Phylogenetic analysis of 17 expanded genes’ CDS sequences in ko01040 pathway from 15 oil plant species, including *A. duranensis*, *A. thaliana*, *B. napus*, *C. avellana*, *C. sinensis*, *G. max*, *J. regia*, *O. davidiana, O. europaea*, *O. intermedia*, *O. nobilis*, *O. sativa*, *S. indicum*, *Z. mays*, and *C. heterophylla*. Seventeen expanded genes of *C. heterophylla* are marked with red stars. **(B)** Genes expression analysis of 17 expanded genes in ko01040 pathway at four successive ovule developmental stages. *C. het*, *C. heterophylla*; BKR, beta-ketoacyl acyl carrier protein reductase; DET, steroid 5-alpha-reductase DET; FAD, fatty acid desaturase; KAT, 3-ketoacyl-CoA thiolase; SAD, stearoyl-acyl carrier protein desaturase.

We previously obtained the transcript sequencing data of hazelnut at four successive ovule developmental stages: ovule formation (stage Ov1), early ovule growth (stage Ov2), rapid ovule growth (stage Ov3), and ovule maturity (Ov4). We reanalyzed the sequencing data using our assembled genome as the reference genome, along with the HazelOmics Database. The transcriptome data were aligned to our assembled genome, and the results showed that 81.48–84.79% reads could be aligned to the genome, indicating that our genome assembly is of good quality and can be used as a reference genome to meet the needs of information analysis (Supplementary Table 18). In the homepage of the HOD database, IDs of the 17 expanded genes were used to search for gene annotation, and these genes encoded fatty acid desaturase (FAD), steroid 5-alpha-reductase DET (DET), 3-ketoacyl-CoA thiolase (KAT), beta-ketoacyl acyl carrier protein reductase (BKR), and stearoyl-acyl carrier protein desaturase (SAD). A phylogenetic tree was further constructed by aligning homologous CDS sequences of these five key enzymes from *C. heterophylla* and 14 other oil plants, showing that 284 sequences were cluster into five clades, four of which (BKR, DET, FAD, and KAT) were relatively conservative while the other one (SAD) with lower conservatism was further divided into different subclusters (Figure 7A). Meanwhile, our interesting 17 expanded genes were evenly distributed in five evolutionary clades consisted with the functional annotation of HOD. Among these, the expression levels of genes encoding FAD, KAT, and SAD were relatively high during the ovule maturity stage, which is consistent with high fatty acid levels at this stage, indicating that they may play an important role in regulating the biosynthesis of unsaturated fatty acids (Figure 7B).

## Discussion

Consistency, integrity, and accuracy are important parameters for evaluating the quality of genome assembly. SMRT sequencing and assembly, followed by error corrections using the Hi-C assisted genome assembly strategy, could effectively improve the genome assembly quality (Jonas et al., 2017). Using this strategy, our sequencing and sequence assembly generated a reference genome of hazelnut with contig N50 of 2.03 Mb. The Hi-C data were then used to cluster 386 contigs into 11 chromosomes with a scaffold N50 of 32.88 Mb, which was consistent with a karyotype of 2n = 22 chromosomes of *C. heterophylla* (Guo et al., 2009). *B. pendula* and *C. avellana* are the only Betulaceae species with available genome information (Pekkinen et al., 2005; Sathuvalli et al., 2011; Chen et al., 2019). Thus, the genome of *C. avellana* plays an important role in interpreting transcriptome data and gene function analysis of hazelnut species. The assembly of the *C. avellana* ‘Jefferson’ genome was based only on the Illumina sequencing approach because of the limitation of technical conditions, and it was found to have a scaffold N50 of 21.5 kb (Sathuvalli et al., 2011). Recently, the chromosome-scale genome of *C. avellana* ‘Tombul’ was assembled, with a total length of 370 Mb and scaffold N50 of 36.65 Mb, using Illumina and Pacbio sequencing (Lucas et al., 2021); furthermore, the chromosome-scale genome of *C. mandshurica* was assembled, with a total length of 368 Mb and scaffold N50 of 14.85 Mb, using Illumina and Nanopore sequencing. Our scaffold N50 was approximately 1,500 times longer than that of ‘Jefferson,’ close to that of *C. avellana* ‘Tombul,’ and much higher than that of *C. mandshurica* (Li et al., 2021). The completeness and high quality of the *C. heterophylla* genome assembled in the present study was further verified by BUSCO alignment (Simao et al., 2015) and Hi-C data mapping (Servant et al., 2015). A total of 94.72% of the test genes could be mapped to the Embryophyta_odb9 database (Simao et al., 2015), of which 92.92% were complete, and LAI was 14.20, indicating a high integrity of the assembled genome. The accuracy of the assembled genome was verified by the homozygous SNP rate of 0.011% and the homozygous indel rate of 0.037%. Thus, the obtained high-quality reference genome of *C. heterophylla* can be beneficial for further molecular breeding and gene function studies of *Corylus* species.

A new *C. heterophylla* genome based on Nanopore long reads was assembled in 2021, with a genome size of 371 Mb and N50 contig size of 2.07 Mb, and 27,591 protein-coding genes were predicted (Zhao et al., 2021). We obtained a 346-Mb genome and predicted 22,319 protein-coding genes. The genome size and number of protein-coding genes predicted in the current study were less than those reported in a previous study (Zhao et al., 2021). Zhao et al. (2021) collected their samples in Yanqing, Beijing, China (40.54°N, 116.06°E), whereas our samples were collected in Yitong (43.34°N, 125.30°E), Jilin, China. The distance between the two locations is more than 800 km, and expectedly, there are differences in the genetic background of these wild plants. Furthermore, these genetic background differences may partly explain the differences in genome size and number of predicted protein-coding genes. Given that hazelnut is a highly heterozygous species and because the accuracy and completeness of our assembly were confirmed, we speculated that some heterozygous sequences were redundant in their assembly, which would lead to redundant genes. N50 contig size was similar for the two assemblies (both ∼2 Mb), but the size of the N90 contig, as determined in the present study, was 424 kb, which was markedly greater than the size of 125 kb for the same contig, as reported by Zhao et al. (2021). These data may suggest that our assembly continuity has notable advantages, and our assembly better avoids the prediction of partial gene. To further investigate genomic conservation and variation, genome-wide collinearity comparison was performed between our self-assembled and previous *C. heterophylla* (Zhao et al., 2021) genomes. Two *C. heterophylla* genomes had extensive collinearity and some differences (Supplementary Figure 1 and Supplementary Table 19). Regions of conserved synteny between our self-assembled and previous *C. heterophylla* genomes shared 19,784 and 21,305 protein-coding genes as well as covered 88.64% and 77.22% of collinearity region, respectively (Supplementary Figure 1). However, owing to the syntenic relationship, the chromosome showed some structural variations (e.g., inversions, translocations, and duplications), and these were mainly located in chromosome 1,2,4,5, and 7 (Supplementary Figure 1). The structural variation and detected mutations, including SNPs, indels, and CNVs (Supplementary Table 19), may suggest the evolutionary differences between our self-assembled and previous *C. heterophylla* (Zhao et al., 2021), which was consistent with the fact that the distance between two sampling locations is more than 800 km.

The *C. heterophylla* phylogenetic tree was constructed using the assembled genome based on SMRT and Hi-C sequencing, and we found that among all related species with available genome information, *C. heterophylla* is a sister group to *C. avellana*, which was consistent with their taxonomic positions and the results of previous phylogenetic analyses (Flora and Sciences, 1979; Cheng et al., 2018a). It was estimated that the divergence time between *C. heterophylla* and *C. avellana* was 11.1 (9.8–13.9) million years ago. On the basis of these results, the expanded and contracted gene families of *C. heterophylla* were further identified. Hazelnuts have a high fatty acid content, and the results of our KEGG enrichment analysis showed significant differences in KEGG pathways of fatty acid elongation and plant–pathogen interaction between *C. heterophylla* and *C. avellana*, suggesting significant differences in the nutrient content of their nuts and in their plant–pathogen interactions. Collectively, these results suggested that fatty acid content and plant–pathogen interactions may be responsible for gene expansion and contraction in *C. heterophylla*.

A database is a comprehensive collection of related data organized for convenient access. As the European hazelnut (*C. avellana*) Genomic Resource Portal (EHG)^4^ only provides a link for the download of data (Sathuvalli et al., 2011; Rowley et al., 2018), which was based on Illumina sequencing of *C. avellana*, it is not a database in the strict sense due to the absence of basic genomic analysis and data mining functions. Based on SMRT sequencing and Hi-C assisted assembly, we established a database of *C. heterophylla*, which is a unique species of *Corylus* from China (Cheng et al., 2019; Liu et al., 2020), with a large distribution area and high biodiversity. Moreover, currently, *C. heterophylla* is the main source of hazelnut products in China. The establishment of our database with mass data will be highly beneficial for promoting the molecular breeding of hazelnut. Our database is the only available genome database of *Corylus* at present. Its function module is simple and clear, and data comparison and mining are convenient and practical. Moreover, different functional modules can be added for database expansion in the future, and the database is convenient for use in studies related to hazelnut.

Fatty acids, which account for 64.48–71.92% of hazelnut kernels (Erdogan and Aygun, 2005), form the most abundant nutrients in hazelnut kernels. FAD, SAD, and KAT are important enzymes involved in the biosynthesis of unsaturated fatty acids in higher plants. FAD catalyzes the formation of double bonds at specific positions of the fatty acid chain and determines the composition and proportion of unsaturated fatty acids (Huang et al., 2018). In *Oryza sativa*, *OsFAD2* is involved in fatty acid desaturation and maintenance of the membrane lipid balance in cells, possibly improving the tolerance of rice to low-temperature stress (Shi et al., 2012). SAD is located in the plastid stroma, catalyzing the desaturation of stearoyl-ACP to oleyl-ACP. SDA determines the ratio of saturated fatty acids and unsaturated fatty acids and is involved in cold acclimation in plants (Li et al., 2015). KAT catalyzes the β-oxidation of fatty acids and is involved in ABA signaling in *Arabidopsis*; furthermore, it is expected to participate in the regulation of plant adaptation to adverse conditions, such as drought and cold stresses (Jiang et al., 2011). Collectively, these genes participating in the biosynthesis of unsaturated fatty acids play an important role in cold resistance in plants. *C. heterophylla* is an endemic species of *Corylus* in China. The proportion of unsaturated fatty acids in the *C. heterophylla* kernel is 94–97%, which is higher than that of *C. avellana* (92–93%) (Song et al., 2008) and much higher than that of most oil plants. *C. heterophylla* is a species with well-known cold-resistant capability, resisting the extreme low winter temperature of −48°C in Northeast China (Chen et al., 2012). In total, 17 expanded genes were found to be significantly enriched in the pathway of unsaturated fatty acid synthesis. Transcriptome analysis at four stages of ovule development showed that the expanded genes of *FAD* (Cor0058010.1), *SAD* (Cor0141290.1), and *KAT* (Cor0122500.1) were highly upregulated at the ovule maturity stage, when fatty acids were most abundant. We deduced that the expansion of *FAD*, *SAD*, and *KAT* may promote high unsaturated fatty acid content in kernels and improve the adaptability of *C. heterophylla* to the cold climate of Northeast China, which may explain why *C. heterophylla* became the dominant *Corylus* species in the area. The important candidate genes for regulating the biosynthesis of unsaturated fatty acids in *C. heterophylla* proposed in this study may also provide a scientific basis for the breeding of hazelnut. In conclusion, our research enhances the understanding of unsaturated fatty acid biosynthesis in hazelnut, and the reference genome and database constructed in this study provide an important platform for future studies on hazelnut and its related species.

## Data Availability Statement

The original contributions presented in the study are publicly available. This data can be found here: Raw sequencing data (PacBio, Illumina, and Hi-C data) for *de novo* whole-genome assembly have been deposited in the NCBI Sequence Read Archive PRJNA664441 (https://www.ncbi.nlm.nih.gov/sra/?term=PRJNA664441). The assembled genome has been deposited in DDBJ/ENA/GenBank under accession number JADFUG000000000. The version described in this article is version JADFUG000000000.

## Author Contributions

JL and YC contributed to study conception and design, collection and/or assembly of data, and data analysis and interpretation. JL and XZ contributed to writing the manuscript. HW, XZ, HH, and DW prepared samples. All authors have read and approved the manuscript.

## Conflict of Interest

The authors declare that the research was conducted in the absence of any commercial or financial relationships that could be construed as a potential conflict of interest.

## Publisher’s Note

All claims expressed in this article are solely those of the authors and do not necessarily represent those of their affiliated organizations, or those of the publisher, the editors and the reviewers. Any product that may be evaluated in this article, or claim that may be made by its manufacturer, is not guaranteed or endorsed by the publisher.

## Footnotes

1
https://hazelnut.data.mocklerlab.org/

2
http://www.timetree.org/

3
http://122.9.151.76/

4
https://hazelnut.data.mocklerlab.org/
