# Supplementary material for: Chromosome-Level Genome Assembly and HazelOmics Database Construction Provides Insights Into Unsaturated Fatty Acid Synthesis and Cold Resistance in Hazelnut (Corylus heterophylla)
Source: Front Plant Sci. 2021 Dec 9;12:766548. doi: 10.3389/fpls.2021.766548 (PMC8695561; doi:10.3389/fpls.2021.766548)
Supplement: Supplementary file 1 [file Data_Sheet_1.DOCX]

Supplementary Material

# Supplementary Data

## Data Availability Statement

Raw sequencing data (PacBio, Illumina, and Hi-C data) for de novo whole-genome assembly have been deposited in the NCBI Sequence Read Archive PRJNA664441 (https://www.ncbi.nlm.nih.gov/sra/?term= PRJNA664441). The assembled genome has been deposited in DDBJ/ENA/GenBank under accession number JADFUG000000000. The version described in this paper is version JADFUG000000000.

# Supplementary Figure

**
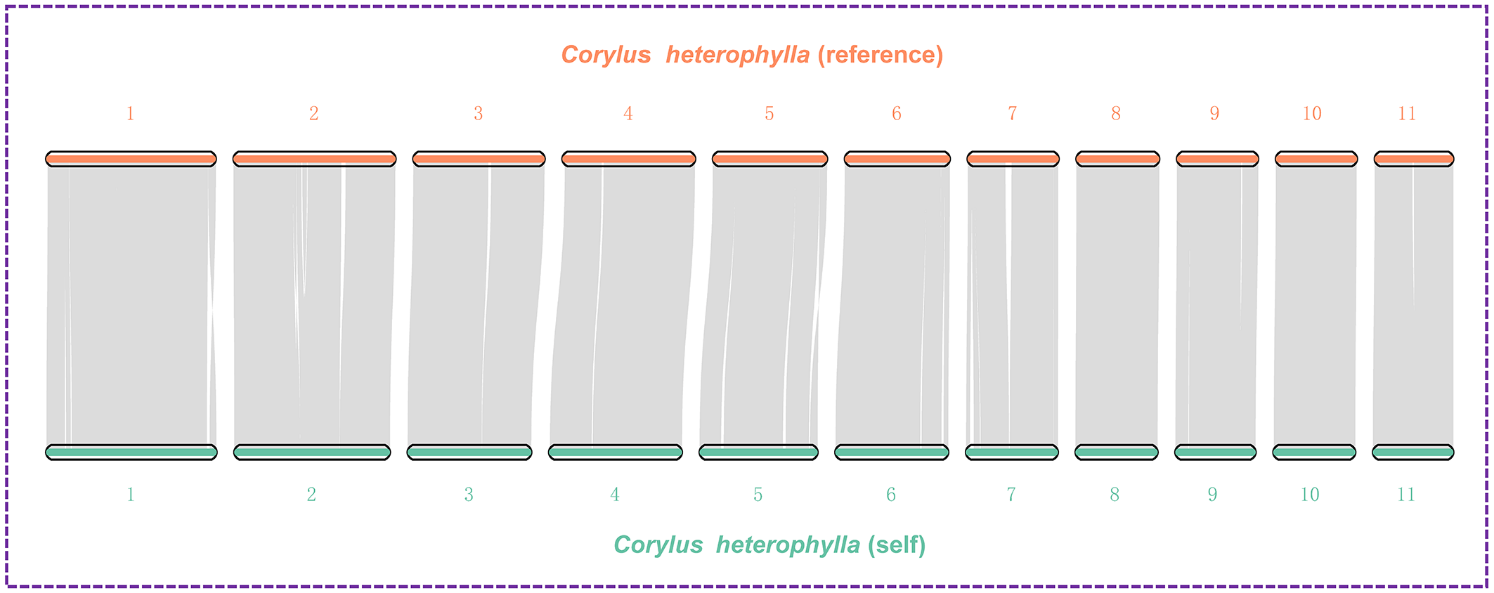
**

**Supplementary Figure 1.** Genome-wide collinearity comparison between self-assembled genome and published genome (GCA 016403345.1)

# Supplementary Tables

**Supplementary Table 1.** Detailed information of all used software in this study

| **Analysis** | **Software** | **Version** | **Function** | **Parameters** |
| --- | --- | --- | --- | --- |
| Genome assembly | Canu | v2.0 | Raw reads assembly | genomeSize=1412m  corOutCoverage=200  correctedErrorRate=0.045  minOverlapLength=500  rawErrorRate=0.300 |
|  | pbmm2 | v1.0.0 | Sequence alignment | - |
|  | arrow | v2.3.3 | Error correction | default |
|  | BWA | v0.7.159 | Sequence alignment | default |
|  | Pilon | v.1.2.2 | Error correction | default |
|  | Purge Haplotigs | - | Redundant sequence removal | --breakfloat 0.1 |
|  | Samtools | v1.7 | Identification of mutations | - |
|  | Picard | v1.128 | Identification of mutations | - |
|  | GATK | v3.3 | Identification of mutations | - |
| Assisted assembly | HiC-Pro | v2.8.0 | Hi-C data processing | - |
|  | JuiceBox | v1.8.8 | Construction of interaction map  and error correction analysis | - |
|  | 3D-DNA | v180922 | 3D-DNA assembly | --enzyme MboI #Hi-C enzyme,{MboI,HindIII,  DpnII}  --length 50 #reads shorter than length will be discarded |
|  | BUSCO | v3.0.1 | Integrity assessment of conserved genes | default |
| Gene annotation | Tandem Repeats Finder | v4.07b | Prediction of tandem repeats | - |
|  | RepeatMasker | v3.3.0 | Annotation of transposons and retrotransposons | - |
|  | RepeatProteinMask | v4.0530 | Annotation of transposons and retrotransposons | - |
|  | RepeatModeler | v1.0.8 | Annotation of interspersed repeats | - |
|  | LTR_FINDER | v1.0.7 | Annotation of interspersed repeats | default |
|  | Augustus | v3.2.2 | Structural annotation | default |
|  | GENSCAN | - | Structural annotation | default |
|  | GlimmerHMM | v3.0.4 | Structural annotation | default |
|  | Tophat alignment | v2.0.13 | RNA-seq data assembly | - |
|  | Cufflinks | v2.2.1 | RNA-seq data assembly | - |
|  | MAKER | - | Prediction of gene sets | -maker_cpu maker 10 -trans_cds_len 150bp |
|  | tRNAscan-SE | v2.0 | Prediction of tRNA | - |
|  | BLASTN | v2.7.1 | Prediction of rRNA | -F F -e 1e-5 -p blastn |
|  | INFERNAL | v1.1 | Prediction of miRNA and snRNA | - |
| Phylogenetic analyses | BLASTP | v2.2.3154 | Sequence alignment | -outfmt 6 -evalue 1e-5 -num_threads 1 |
|  | Orthofinder | v2.2.7 | Screening of homologous low copy genes | default, Copy number < = 4 |
|  | OrthoMCL | v2.0.9 | Evaluation of gene family membership | -i identity 30 ;-2 coverage 50 |
|  | MCL | v14-137 | Evaluation of gene family membership | default |
|  | Muscle | v3.8.31 | Sequence alignment | - |
|  | RAxML | v8.2.1156 | Construction of phylogenetic tree | -f a -x 12345 N 100 -p 12345 -m GTRGAMMA |
|  | r8s | v1.71 | Estimation of divergence time | -b |
|  | MCMCTREE | v4.4 | Estimation of divergence time | seed = -1, ndata = 1, model = 0 |
|  | CAFE | v4.2.1 | Expansion or contraction analysis | default |
| Construction of HOD | Vue.JS framework | - | Website interface | - |
|  | Spring Boot | - | Database server development | - |
|  | JDK8 | - | Database server development | - |
|  | MySQL | - | Database server development | - |
|  | JBrowse | 1.15.3- release | Data visualization | - |
|  | BLAST | - | Sequence query option | - |
|  | Primer3 | - | Primer design | - |
| RNA-seq analysis | HISAT2 | v2.1.0 | Reference genome alignment | --novel-splicesite-outfile XX.ss |
|  | Samtools | v1.9 | Sam format to BAM | - |
|  | qualimap | v2.2.1 | Statistical analysis | rnaseq |
|  | StringTie | 2.1.3b. Linux_x86_64 | Transcriptional assembly and  gene expression analysis | -f 0.2 -a 30 -c 5 -j 5 --merge -e -p 4 |
|  | Transdecoder | v5.0.2 | Predict ORF | - |
|  | KOBAS | v3.0 | KEGG enrichment analysis | - |

**Supplementary Table 2.** Detailed information of all downloaded sequences in this study

| **Species** | **Version** | **Database** | **Website** |
| --- | --- | --- | --- |
| *O. sativa* | GCF_001433935.1 | NCBI |  |
| *A. thaliana* | GCF_000001735.3 | NCBI |  |
| *C. avellane_*Jefferson | - | European Hazelnut  Genomic Resource Portal | https://hazelnut.data.mocklerlab.org |
| *C. avellane_*Tombul | 2021/2/29 | Hardwood Genomics Project | https://www.hardwoodgenomics.org/Genome-assembly/4302786?tripal_pane=group_downloads |
| *C. heterophylla_*ref | GCA_016403345.1 | NCBI |  |
| *C. mandshurica* | 2020/6/21 | figshare | https://figshare.com/articles/dataset/Corylus_mandshurica_genome/12523124 |
| *B. pendula* | GCA_900184695.1 | NCBI |  |
| *J. regia* | GCF_001411555.1 | NCBI |  |
| *C. mollissima* | 2019/8/6 | GigaDB | ftp://parrot.genomics.cn/gigadb/pub/10.5524/100001_101000/100643/ |
| *P. euphratica* | GCF_000495115.1 | NCBI |  |
| *P. alba* | GCA_005239225.1 | NCBI |  |
| *P. trichocarpa* | GCF_000002775.4 | NCBI |  |
| *S. brachista* | GCA_009078335.1 | NCBI |  |
| *M. esculenta* | GCF_001659605.1 | NCBI |  |
| *H. brasiliensis* | GCF_001654055.1 | NCBI |  |
| *R. communis* | GCF_000151685.1 | NCBI |  |
| *P. persica* | GCF_000346465.2 | NCBI |  |
| *A. duranensis* | GCF_000817695.2 | NCBI |  |
| *O. europaea* | GCA_002742605.1 | NCBI |  |
| *C. sinensis* | GCF_004153795.1 | NCBI |  |
| *S. indicum* | GCF_000512975.1 | NCBI |  |
| *G. max* | Wm82.a2.v1 | Phytozome |  |
| *Z. mays* | ZmaysPH207 v1.1 | Phytozome |  |
| *B. napus* | GCF_000686985.1 | NCBI |  |
| *O. intermedia* | 2020/10/9 | figshare | <https://figshare.com/articles/dataset/Genomes_of_Ostryopsis_species/13060280> |
| *O. nobilis* | 2020/10/9 | figshare | https://figshare.com/articles/dataset/Genomes_of_Ostryopsis_species/13060280 |
| *O. davidiana* | 2020/10/9 | figshare | https://figshare.com/articles/dataset/Genomes_of_Ostryopsis_species/13060280 |

**Supplementary Table 3.** Partial blast results with NT database

| **Genus** | **Blast number** | **Total blast number** | **Total (%)** |
| --- | --- | --- | --- |
| *Juglans* | 15,685 | 29,800 | 52.63 |
| *Corylus* | 3,040 | 29,800 | 10.20 |
| *Vitis* | 2,183 | 29,800 | 7.33 |
| *Theobroma* | 832 | 29,800 | 2.79 |
| *Cornus* | 791 | 29,800 | 2.65 |

**Supplementary Table 4.** Evaluating of genome assembly by BUSCO

| **Type** | **Proteins** | **Percentage (%)** |
| --- | --- | --- |
| Complete BUSCOs (C) | 1,338 | 92.92 |
| Complete and single-copy BUSCOs (S) | 1,263 | 87.71 |
| Complete and duplicated BUSCOs (D) | 75 | 5.21 |
| Fragmented BUSCOs (F) | 26 | 1.81 |
| Missing BUSCOs (M) | 76 | 5.28 |
| Total BUSCO groups searched | 1,440 | 100.00 |

**Supplementary Table 5.** Statistical results of quality control of genome sequencing

| **Assembly** | **Size (bp)** |
| --- | --- |
| Raw reads number | 392,233,610 |
| Raw Bases(bp) | 58,835,041,500 |
| Clean reads number | 383,274,912 |
| Clean bases (bp) | 56,648,423,236 |
| Read length (bp) | 150 |
| Q20 (%) | 96.24 |
| Q30 (%) | 90.4 |
| GC Content (%) | 36.84 |

**Supplementary Table 6.** Sequences alignment with assembled genome

|  | **Read1** | **Read2** |
| --- | --- | --- |
| Unique Alignments | 122,123,789 | 115,537,222 |
| Multiple Alignments | 37,405,895 | 41,106,530 |
| Failed to Align | 26,717,578 | 29,707,168 |
| Paired | 84,085,237 | 84,085,237 |

**Supplementary Table 7.** Statistics results after sequences filtering

|  | **Di-Tag Count** | **Percent in Paired (%)** | **Percent in Total Reads (%)** |
| --- | --- | --- | --- |
| Same Circularised | 81,765 | 0.1 | 0.04 |
| Same Fragment Dangling Ends | 204,624 | 0.24 | 0.11 |
| Same Fragment Internal | 1,214,478 | 1.44 | 0.63 |
| Re-ligation | 1,582,039 | 1.88 | 0.83 |
| Contiguous Sequence | 0 | 0 | 0 |
| Wrong Size | 0 | 0 | 0 |
| Invalid Pairs | 3,082,906 | 3.67 | 1.61 |
| Valid Pairs | 81,002,331 | 96.33 | 42.27 |
| Valid Pairs (de-duplication) | 61,989,951 | 73.72 | 32.35 |

**Supplementary Table 8.** Statistics results of homozygous and heterozygous rate

| **Sample** | **Homozygous SNP rate (%)** | **Homozygous InDel rate (%)** | **Heterozygous SNP rate (%)** | **Heterozygous InDel rate (%)** |
| --- | --- | --- | --- | --- |
| *Corylus heterophylla* | 0.011 | 0.037 | 1.118 | 0.216 |

**Supplementary Table 9.** Integrity assessment after *C. heterophylla* genome assembly

| **Chromosome** | **Start site** | **End site** | **Intact LTR rate (%)** | **Total LTR rate (%)** | **Raw LAI** | **LAI** |
| --- | --- | --- | --- | --- | --- | --- |
| Whole_genome | 1 | 346,578,452 | 1.91 | 22.28 | 8.57 | 14.20 |

Note: Raw LAI = (Intact LTR sequence length/Total LTR sequence length) * 100, LAI = Raw LAI + 2.8138 × (94 – whole genome LTR identity)

**Supplementary Table 10.** Statistics for gene prediction in *C. heterophylla* genome

| **Gene set** | **Protein coding gene number** | **Average gene length (bp)** | **Average CDS length (bp)** | **Average exon per gene** | **Average exon length (bp)** | **Average intron length (bp)** |
| --- | --- | --- | --- | --- | --- | --- |
| denovo/GlimmmerHMM | 32,600 | 9,836 | 1,545 | 4.62 | 334.57 | 2,292 |
| denovo/AUGUSTUS | 10,117 | 3,338 | 866.92 | 4.08 | 212.44 | 801.95 |
| homo/*Abrus_precatorius* | 79,912 | 20,308 | 964.55 | 3.28 | 294.45 | 8,499 |
| homo/*Aegilops_tauschii_*subsp_tauschii | 81,541 | 20,833 | 972.61 | 3.07 | 316.78 | 9,593 |
| homo/*Amborella_trichopoda* | 75,486 | 16,566 | 795.62 | 2.94 | 270.6 | 8,128 |
| homo/*Carpinus_fangiana* | 75,666 | 15,600 | 787.21 | 3.16 | 249.22 | 6,862 |
| homo/*Castanea_mollissima* | 95,412 | 13,844 | 751.38 | 2.71 | 277.17 | 7,653 |
| homo/*Corylus_avellane*_Jefferson | 88,630 | 6,016 | 384.32 | 1.93 | 199.03 | 6,049 |
| homo/*Juglans_regia* | 95,829 | 19,833 | 1,000 | 3.08 | 324.23 | 9,034 |
| homo/*Quercus_suber* | 94,667 | 21,876 | 1,045 | 3.18 | 328.97 | 9,573 |
| trans.orf/RNAseq | 11,618 | 7,446 | 1,456 | 7.30 | 305.31 | 828.43 |
| BUSCO | 1,481 | 6,048 | 1,569 | 9.27 | 169.29 | 541.55 |
| MAKER | 21,332 | 8,810 | 1,126 | 5.41 | 351.54 | 1,567 |
| HiCESAP | 22,319 | 6,646 | 1,201 | 5.61 | 330.23 | 1,040 |

**Supplementary Table 11.** Category of repeat sequences in *C. heterophylla* genome

|  | ≥ **20% overlap** | |  | ≥ **50% overlap** | |  | ≥ **80% overlap** | |
| --- | --- | --- | --- | --- | --- | --- | --- | --- |
|  | No. | Ratio (%) |  | No. | Ratio (%) |  | No. | Ratio (%) |
| **P (single)** | 125 | 0.56 |  | 475 | 2.13 |  | 1,423 | 6.38 |
| **P (more)** | 47 | 0.21 |  | 69 | 0.31 |  | 93 | 0.42 |
| **H (single)** | 177 | 0.79 |  | 252 | 1.13 |  | 476 | 2.13 |
| **H (more)** | 715 | 3.2 |  | 987 | 4.42 |  | 1,434 | 6.43 |
| **C (single)** | 35 | 0.16 |  | 62 | 0.28 |  | 197 | 0.88 |
| **C (more)** | 0 | 0 |  | 0 | 0 |  | 0 | 0 |
| **PH** | 4,454 | 19.96 |  | 4,374 | 19.6 |  | 3,504 | 15.7 |
| **PC** | 115 | 0.52 |  | 99 | 0.44 |  | 192 | 0.86 |
| **HC** | 1,888 | 8.46 |  | 3,127 | 14.01 |  | 6,268 | 28.08 |
| **PHC** | 14,763 | 66.15 |  | 12,872 | 57.67 |  | 8,657 | 38.79 |

**Supplementary Table 12.** Annotation statistics for *C. heterophylla* genome

| **Terms** | **Database** | **Number** | **Percentage (%)** | **Total number** | **Total percentage (%)** |
| --- | --- | --- | --- | --- | --- |
| Total protein |  |  |  | 22,319 | 100 |
| Annotated | InterPro | 17,543 | 78.6 | 21,056 | 94.34 |
|  | GO | 12,015 | 53.83 |  |  |
|  | KEGG-ALL | 20,732 | 92.89 |  |  |
|  | KEGG-KO | 8,151 | 36.52 |  |  |
|  | Swissprot | 15,017 | 67.28 |  |  |
|  | TrEMBL | 20,977 | 93.99 |  |  |
|  | TF | 1,376 | 6.17 |  |  |
|  | Pfam | 17,165 | 76.91 |  |  |
|  | NR | 20,916 | 93.71 |  |  |
|  | KOG | 17,841 | 79.94 |  |  |
| Unannotated |  |  |  | 1,263 | 5.66 |

**Supplementary Table 13.** Annotation evaluation by BUSCO

| **Type** | **Assembly** | | | **Annotation** | |  |
| --- | --- | --- | --- | --- | --- | --- |
|  | Proteins | Percentage (%) | Proteins | | Percentage (%) | |
| Complete BUSCOs | 1,334 | 92.7 | 1,341 | | 93.1 | |
| Complete Single-Copy BUSCOs | 1,258 | 87.4 | 1,269 | | 88.1 | |
| Complete Duplicated BUSCOs | 76 | 5.3 | 72 | | 5.0 | |
| Fragmented BUSCOs | 30 | 2.1 | 37 | | 2.6 | |
| Missing BUSCOs | 76 | 5.2 | 62 | | 4.3 | |
| Total BUSCO groups searched | 1,440 | 100.0 | 1,440 | | 100.0 | |

**Supplementary Table 14.** Genes cluster summarization of *C. heterophylla* and other closely related species

| **Species** | **Genes number** | **Unclustered genes** | **Genes in families** | **Family number** | **Unique families** | **Unique families genes** | **Common families** | **Common families genes** | **Single copy** | **Single copy genes** | **Average genes per family** |
| --- | --- | --- | --- | --- | --- | --- | --- | --- | --- | --- | --- |
| *C. heterophylla* | 22,319 | 2,636 | 19,683 | 14,421 | 113 | 409 | 6,504 | 10,044 | 695 | 695 | 1.365 |
| *O. sativa Japonica* | 28,314 | 4,834 | 23,480 | 12,160 | 1,230 | 4,843 | 6,504 | 11,785 | 695 | 695 | 1.931 |
| *A. thaliana* | 27,298 | 4,037 | 23,261 | 12,980 | 753 | 3,005 | 6,504 | 11,728 | 695 | 695 | 1.792 |
| *C. avellana*_ Tombul | 27,270 | 4,209 | 23,061 | 14,577 | 414 | 1,105 | 6,504 | 11,463 | 695 | 695 | 1.582 |
| *C. mandshurica* | 28,409 | 4,552 | 23,857 | 16,017 | 469 | 1,352 | 6,504 | 10,969 | 695 | 695 | 1.489 |
| *B. pendula* | 26,069 | 5,705 | 20,364 | 14,476 | 342 | 862 | 6,504 | 10,234 | 695 | 695 | 1.407 |
| *J. regia* | 33,899 | 3,823 | 30,076 | 14,765 | 551 | 1,687 | 6,504 | 14,335 | 695 | 695 | 2.037 |
| *C. mollissima* | 36,479 | 5,631 | 30,848 | 15,837 | 1,074 | 3,963 | 6,504 | 12,062 | 695 | 695 | 1.948 |
| *P. euphratica* | 29,950 | 1,234 | 28,716 | 15,685 | 65 | 142 | 6,504 | 15,023 | 695 | 695 | 1.831 |
| *P. alba* | 32,460 | 3,683 | 28,777 | 15,883 | 221 | 592 | 6,504 | 14,646 | 695 | 695 | 1.812 |
| *P. trichocarpa* | 31,205 | 1,384 | 29,821 | 15,915 | 57 | 139 | 6,504 | 15,256 | 695 | 695 | 1.874 |
| *S. brachista* | 30,154 | 3,880 | 26,274 | 14,831 | 401 | 1,172 | 6,504 | 13,351 | 695 | 695 | 1.772 |
| *M. esculenta* | 27,772 | 2,035 | 25,737 | 14,506 | 130 | 555 | 6,504 | 13,338 | 695 | 695 | 1.774 |
| *H. brasiliensis* | 33,842 | 3,474 | 30,368 | 15,136 | 288 | 937 | 6,504 | 15,310 | 695 | 695 | 2.006 |
| *R. communis* | 19,496 | 1,205 | 18,291 | 13,969 | 70 | 209 | 6,504 | 9,620 | 695 | 695 | 1.309 |
| *P. persica* | 22,927 | 1,351 | 21,576 | 14,005 | 273 | 1,235 | 6,504 | 10,527 | 695 | 695 | 1.541 |

**Supplementary Table 15.** Genes cluster summarization of *C. heterophylla* and other oil plant species

| **Species** | **Genes number** | **Unclustered genes** | **Genes in families** | **Family number** | **Unique families** | **Unique families genes** | **Common families** | **Common families genes** | **Single copy genes** | **Average**  **Genes**  **Per**  **family** |
| --- | --- | --- | --- | --- | --- | --- | --- | --- | --- | --- |
| *O. sativa* | 28,727 | 17,179 | 4,069 | 4,259 | 6,173 | 12,651 | 4 | 4 | 1.672 | 1.672 |
| *A. duranensis* | 36,734 | 22,590 | 10,023 | 10,659 | 6,173 | 13,185 | 4 | 4 | 1.626 | 1.626 |
| *J. regia* | 30,703 | 14,042 | 1,159 | 1,245 | 6,173 | 15,542 | 4 | 4 | 2.187 | 2.187 |
| *O. europaea* | 40,119 | 17,444 | 4,180 | 4,544 | 6,173 | 18,188 | 4 | 4 | 2.3 | 2.3 |
| *C. sinensis* | 50,831 | 23,453 | 9,437 | 10,497 | 6,173 | 18,470 | 4 | 4 | 2.167 | 2.167 |
| *S. indicum* | 24,068 | 13,268 | 880 | 973 | 6,173 | 12,897 | 4 | 4 | 1.814 | 1.814 |
| *G. max* | 73,320 | 23,885 | 10,000 | 10,341 | 6,173 | 36,056 | 4 | 4 | 3.07 | 3.07 |
| *Z. mays* | 40,557 | 23,475 | 9,769 | 10,077 | 6,173 | 15,655 | 4 | 4 | 1.728 | 1.728 |
| *A. thaliana* | 38,931 | 17,999 | 3,772 | 3,841 | 6,173 | 17,560 | 4 | 4 | 2.163 | 2.163 |
| *C. avellana* | 28,167 | 22,563 | 11,981 | 12,009 | 6,173 | 9,979 | 4 | 4 | 1.248 | 1.248 |
| *B. napus* | 96,958 | 29,009 | 14,125 | 15,825 | 6,173 | 40,900 | 4 | 4 | 3.342 | 3.342 |
| *O. intermedia* | 24,790 | 15,994 | 580 | 580 | 6,173 | 11,102 | 4 | 4 | 1.55 | 1.55 |
| *O. nobilis* | 24,634 | 15,818 | 753 | 755 | 6,173 | 11,110 | 4 | 4 | 1.557 | 1.557 |
| *O. davidiana* | 25,184 | 15,994 | 598 | 602 | 6,173 | 11,233 | 4 | 4 | 1.575 | 1.575 |
| *C. heterophylla* | 22,319 | 14,584 | 1,272 | 1,277 | 6,173 | 10,655 | 4 | 4 | 1.53 | 1.53 |

**Supplementary Table 16.** KEGG enrichment of contracted genes in *C. heterophylla*

| **KEGG Pathway** | **Pathway ID** | **Gene Number** | **Background number** | **P-Value** | **Q-Value** |
| --- | --- | --- | --- | --- | --- |
| Glutathione metabolism | ko00480 | 14 | 76 | 2.56E-10 | 1.10E-08 |
| Flavone and flavonol biosynthesis | ko00944 | 2 | 3 | 1.39E-05 | 4.49E-04 |
| Linoleic acid metabolism | ko00591 | 3 | 13 | 1.99E-04 | 4.93E-03 |
| Pancreatic secretion | ko04972 | 4 | 24 | 2.29E-04 | 4.93E-03 |
| Collecting duct acid secretion | ko04966 | 4 | 25 | 2.81E-04 | 5.18E-03 |
| TGF-beta signaling pathway | ko04350 | 4 | 26 | 3.41E-04 | 5.50E-03 |
| Tyrosine metabolism | ko00350 | 5 | 42 | 4.69E-04 | 6.05E-03 |
| Naphthalene degradation | ko00626 | 2 | 7 | 4.53E-04 | 6.05E-03 |
| Caffeine metabolism | ko00232 | 1 | 2 | 5.81E-04 | 6.61E-03 |
| Riboflavin metabolism | ko00740 | 3 | 17 | 6.15E-04 | 6.61E-03 |
| Degradation of aromatic compounds | ko01220 | 2 | 9 | 1.05E-03 | 9.35E-03 |
| Osteoclast differentiation | ko04380 | 3 | 19 | 9.65E-04 | 9.35E-03 |
| NOD-like receptor signaling pathway | ko04621 | 5 | 49 | 1.09E-03 | 9.35E-03 |
| Endocrine and other factor-regulated calcium reabsorption | ko04961 | 3 | 21 | 1.43E-03 | 1.16E-02 |
| Plant hormone signal transduction | ko04075 | 12 | 210 | 1.69E-03 | 1.29E-02 |
| Isoquinoline alkaloid biosynthesis | ko00950 | 3 | 23 | 2.04E-03 | 1.39E-02 |
| Mineral absorption | ko04978 | 2 | 11 | 1.99E-03 | 1.39E-02 |
| Retinol metabolism | ko00830 | 2 | 12 | 2.60E-03 | 1.68E-02 |
| Thiamine metabolism | ko00730 | 3 | 26 | 3.26E-03 | 1.98E-02 |
| Fat digestion and absorption | ko04975 | 1 | 4 | 3.38E-03 | 1.98E-02 |
| alpha-Linolenic acid metabolism | ko00592 | 4 | 44 | 3.96E-03 | 2.22E-02 |
| Lysosome | ko04142 | 5 | 64 | 4.32E-03 | 2.23E-02 |
| Arachidonic acid metabolism | ko00590 | 2 | 14 | 4.16E-03 | 2.23E-02 |
| GABAergic synapse | ko04727 | 3 | 29 | 4.90E-03 | 2.43E-02 |
| Chloroalkane and chloroalkene degradation | ko00625 | 2 | 15 | 5.10E-03 | 2.44E-02 |
| Circadian rhythm | ko04710 | 3 | 30 | 5.54E-03 | 2.55E-02 |
| Vasopressin-regulated water reabsorption | ko04962 | 3 | 32 | 7.01E-03 | 3.12E-02 |
| Quorum sensing | ko02024 | 4 | 52 | 8.11E-03 | 3.20E-02 |
| Autophagy - other eukaryotes | ko04136 | 3 | 33 | 7.83E-03 | 3.20E-02 |
| Steroid hormone biosynthesis | ko00140 | 1 | 6 | 8.18E-03 | 3.20E-02 |
| Long-term depression | ko04730 | 1 | 6 | 8.18E-03 | 3.20E-02 |
| Hippo signaling pathway - multiple species | ko04392 | 1 | 7 | 1.13E-02 | 4.15E-02 |
| Anthocyanin biosynthesis | ko00942 | 1 | 7 | 1.13E-02 | 4.15E-02 |
| Mitophagy - animal | ko04137 | 3 | 37 | 1.17E-02 | 4.20E-02 |
| Purine metabolism | ko00230 | 8 | 156 | 1.32E-02 | 4.60E-02 |
| Hedgehog signaling pathway - fly | ko04341 | 3 | 39 | 1.41E-02 | 4.68E-02 |
| Pentose and glucuronate interconversions | ko00040 | 5 | 82 | 1.41E-02 | 4.68E-02 |
| Synaptic vesicle cycle | ko04721 | 4 | 60 | 1.46E-02 | 4.71E-02 |

**Supplementary Table 17.** KEGG enrichment of expanded genes in *C. heterophylla*

| **KEGG Pathway** | **Pathway**  **ID** | **Gene Number** | **Background number** | **P-Value** | **Q-Value** |
| --- | --- | --- | --- | --- | --- |
| Glycosphingolipid biosynthesis - lacto and neolacto series | ko00601 | 3 | 3 | 0.00E+00 | 0.00E+00 |
| Tropane, piperidine and pyridine alkaloid biosynthesis | ko00960 | 19 | 29 | 1.51E-11 | 1.05E-09 |
| Plant-pathogen interaction | ko04626 | 57 | 171 | 1.14E-11 | 1.05E-09 |
| Estrogen signaling pathway | ko04915 | 22 | 40 | 1.10E-10 | 5.70E-09 |
| Monoterpenoid biosynthesis | ko00902 | 14 | 19 | 2.53E-10 | 1.05E-08 |
| Longevity regulating pathway - multiple species | ko04213 | 26 | 60 | 3.89E-09 | 1.35E-07 |
| Biosynthesis of unsaturated fatty acids | ko01040 | 17 | 30 | 4.72E-09 | 1.40E-07 |
| Toll and Imd signaling pathway | ko04624 | 22 | 47 | 7.56E-09 | 1.97E-07 |
| Toll-like receptor signaling pathway | ko04620 | 20 | 42 | 2.10E-08 | 4.85E-07 |
| MAPK signaling pathway | ko04010 | 21 | 46 | 2.74E-08 | 5.71E-07 |
| Neurotrophin signaling pathway | ko04722 | 28 | 74 | 4.71E-08 | 8.90E-07 |
| NF-kappa B signaling pathway | ko04064 | 20 | 45 | 9.74E-08 | 1.69E-06 |
| Jak-STAT signaling pathway | ko04630 | 7 | 8 | 1.27E-07 | 2.03E-06 |
| Antigen processing and presentation | ko04612 | 21 | 56 | 1.88E-06 | 2.79E-05 |
| ErbB signaling pathway | ko04012 | 7 | 10 | 4.41E-06 | 6.12E-05 |
| Melanogenesis | ko04916 | 8 | 14 | 1.81E-05 | 2.35E-04 |
| Glycosphingolipid biosynthesis - globo and isoglobo series | ko00603 | 6 | 9 | 2.59E-05 | 3.16E-04 |
| Bisphenol degradation | ko00363 | 5 | 7 | 4.18E-05 | 4.57E-04 |
| Polycyclic aromatic hydrocarbon degradation | ko00624 | 5 | 7 | 4.18E-05 | 4.57E-04 |
| Novobiocin biosynthesis | ko00401 | 4 | 5 | 4.93E-05 | 5.12E-04 |
| T cell receptor signaling pathway | ko04660 | 7 | 14 | 1.76E-04 | 1.74E-03 |
| Fatty acid metabolism | ko01212 | 20 | 68 | 2.07E-04 | 1.95E-03 |
| Thyroid hormone signaling pathway | ko04919 | 12 | 33 | 2.36E-04 | 2.14E-03 |
| Cytosolic DNA-sensing pathway | ko04623 | 10 | 27 | 5.07E-04 | 4.40E-03 |
| RNA polymerase | ko03020 | 14 | 45 | 6.69E-04 | 5.57E-03 |
| Signaling pathways regulating pluripotency of stem cells | ko04550 | 4 | 7 | 8.12E-04 | 5.82E-03 |
| Phosphatidylinositol signaling system | ko04070 | 16 | 55 | 8.05E-04 | 5.82E-03 |
| Phenylpropanoid biosynthesis | ko00940 | 38 | 170 | 7.40E-04 | 5.82E-03 |
| Other types of O-glycan biosynthesis | ko00514 | 4 | 7 | 8.12E-04 | 5.82E-03 |
| Calcium signaling pathway | ko04020 | 11 | 33 | 9.57E-04 | 6.50E-03 |
| Renin secretion | ko04924 | 7 | 17 | 9.69E-04 | 6.50E-03 |
| Mitophagy - yeast | ko04139 | 13 | 44 | 1.67E-03 | 1.07E-02 |
| Ubiquitin mediated proteolysis | ko04120 | 28 | 122 | 1.91E-03 | 1.07E-02 |
| Fatty acid biosynthesis | ko00061 | 12 | 40 | 1.96E-03 | 1.07E-02 |
| Gastric acid secretion | ko04971 | 4 | 8 | 1.92E-03 | 1.07E-02 |
| Prolactin signaling pathway | ko04917 | 4 | 8 | 1.92E-03 | 1.07E-02 |
| Circadian rhythm - fly | ko04711 | 4 | 8 | 1.92E-03 | 1.07E-02 |
| Phototransduction - fly | ko04745 | 4 | 8 | 1.92E-03 | 1.07E-02 |
| Th17 cell differentiation | ko04659 | 6 | 15 | 2.17E-03 | 1.15E-02 |
| Isoquinoline alkaloid biosynthesis | ko00950 | 8 | 23 | 2.30E-03 | 1.18E-02 |
| B cell receptor signaling pathway | ko04662 | 7 | 19 | 2.33E-03 | 1.18E-02 |
| Nitrogen metabolism | ko00910 | 10 | 32 | 2.61E-03 | 1.29E-02 |
| Glucosinolate biosynthesis | ko00966 | 4 | 9 | 3.83E-03 | 1.81E-02 |
| Salivary secretion | ko04970 | 4 | 9 | 3.83E-03 | 1.81E-02 |
| Protein processing in endoplasmic reticulum | ko04141 | 39 | 195 | 5.59E-03 | 2.58E-02 |
| Aldosterone synthesis and secretion | ko04925 | 4 | 10 | 6.80E-03 | 3.07E-02 |
| Aminobenzoate degradation | ko00627 | 5 | 14 | 7.54E-03 | 3.34E-02 |
| Anthocyanin biosynthesis | ko00942 | 3 | 7 | 8.87E-03 | 3.84E-02 |
| Gap junction | ko04540 | 6 | 19 | 1.03E-02 | 4.37E-02 |
| Limonene and pinene degradation | ko00903 | 5 | 15 | 1.11E-02 | 4.54E-02 |
| Glucagon signaling pathway | ko04922 | 12 | 48 | 1.10E-02 | 4.54E-02 |

**Supplementary Table 18.** Statistics of transcriptome alignment efficiency

| **Sample** | **Not aligned** | **Reads aligned** |
| --- | --- | --- |
| Ov1A | 19788031(18.50%) | 87151837(81.50%) |
| Ov1B | 19995169(18.70%) | 86928495(81.30%) |
| Ov1C | 20855110(18.52%) | 91772440(81.48%) |
| Ov2A | 16760177(15.44%) | 91802117(84.56%) |
| Ov2B | 16616602(15.23%) | 92477430(84.77%) |
| Ov2C | 16298326(15.21%) | 90833124(84.79%) |
| Ov3A | 17846040(16.50%) | 90285130(83.50%) |
| Ov3B | 18921053(17.24%) | 90853607(82.76%) |
| Ov3C | 17881848(16.57%) | 90024618(83.43%) |
| Ov4A | 18733876(16.93%) | 91921312(83.07%) |
| Ov4B | 18730388(17.43%) | 88707276(82.57%) |
| Ov4C | 19776944(18.20%) | 88872382(81.80%) |

Note: Ovules samples were from four developmental stages, namely, ovule formation (Ov1), early ovule growth (Ov2), rapid ovule growth (Ov3), and ovule maturity (Ov4). In the sample name, the capital letters A, B and C after the numbers indicate three biological replicates. Not aligned: the percentage of reads not aligned with assembled genome; reads aligned: the percentage of reads aligned to assembled genome.

**Supplementary Table 19.** Statistics of SyRi variation detection results

| **Annotation_type** | **Variation_type** | **Count** | **Length_ reference** | **Length_ query** |
| --- | --- | --- | --- | --- |
| Structural annotations | Syntenic regions | 3,780 | 259,780,058 | 249,667,087 |
|  | Inversions | 98 | 13,647,474 | 13,124,635 |
|  | Translocations | 2,089 | 12,725,018 | 12,720,794 |
|  | Duplications (reference) | 553 | 3,414,299 | - |
|  | Duplications (query) | 3,841 | - | 13,500,068 |
|  | Not aligned (reference) | 4,956 | 55,215,480 | - |
|  | Not aligned (query) | 8,838 | - | 54,194,508 |
| Sequence annotations | SNPs | 2,283,021 | 2,283,021 | 2,283,021 |
|  | Insertions | 556,960 | - | 3,122,009 |
|  | Deletions | 163,973 | 2,822,976 | - |
|  | Copygains | 173 | - | 1,395,253 |
|  | Copylosses | 626 | 3,160,730 | - |
|  | Highly diverged | 22,128 | 50,184,703 | 40,735,073 |
|  | Tandem repeats | 27 | 53,806 | 53,284 |

Note: Structural annotations: annotation results of structural variation; Sequence annotations: annotation results for specific sequence variation; Reference is the published genome of NCBI (GCA 016403345.1), and query is the self-assembled genome.
